# Sorghum *MSD3* Encodes an ω-3 Fatty Acid Desaturase that Increases Grain Number by Reducing Jasmonic Acid Levels

**DOI:** 10.3390/ijms20215359

**Published:** 2019-10-28

**Authors:** Lavanya Dampanaboina, Yinping Jiao, Junping Chen, Nicholas Gladman, Ratan Chopra, Gloria Burow, Chad Hayes, Shawn A. Christensen, John Burke, Doreen Ware, Zhanguo Xin

**Affiliations:** 1 Plant Stress and Germplasm Development Unit, Cropping Systems Research Laboratory, U.S. Department of Agriculture-Agricultural Research Service, Lubbock, TX 79415, USA; lavanya.dampanaboina@ttu.edu (L.D.); yjiao@cshl.edu (Y.J.); Junping.chen@ars.usda.gov (J.C.); gladman@cshl.edu (N.G.); rchopra@umn.edu (R.C.); Gloria.burow@ars.usda.gov (G.B.); Chad.Hayes@ars.usda.gov (C.H.); John.Burke@ars.usda.gov (J.B.); 2 Cold Spring Harbor Laboratory, Cold Spring Harbor, New York, NY 11724, USA; 3 Current address: Department of Agronomy and Plant Genetics, University of Minnesota, St. Paul, MN 55108, USA; 4 Chemistry Research Unit, USDA-ARS, 1700 S.W. 23rd Drive, Gainesville, FL 32608, USA; Shawn.Christensen@ars.usda.gov; 5 U.S. Department of Agriculture-Agricultural Research Service, NEA Robert W. Holley Center for Agriculture and Health, Cornell University, Ithaca, New York, NY 14853, USA

**Keywords:** jasmonic acid, fatty acid desaturase, multiseeded, *msd*, grain number, MutMap, sorghum

## Abstract

Grain number per panicle is an important component of grain yield in sorghum (*Sorghum bicolor* (L.)) and other cereal crops. Previously, we reported that mutations in multi-seeded 1 (*MSD1)* and *MSD2* genes result in a two-fold increase in grain number per panicle due to the restoration of the fertility of the pedicellate spikelets, which invariably abort in natural sorghum accessions. Here, we report the identification of another gene, *MSD3,* which is also involved in the regulation of grain numbers in sorghum. Four bulked F_2_ populations from crosses between BTx623 and each of the independent *msd* mutants p6, p14, p21, and p24 were sequenced to 20× coverage of the whole genome on a HiSeq 2000 system. Bioinformatic analyses of the sequence data showed that one gene, Sorbi_3001G407600, harbored homozygous mutations in all four populations. This gene encodes a plastidial ω-3 fatty acid desaturase that catalyzes the conversion of linoleic acid (18:2) to linolenic acid (18:3), a substrate for jasmonic acid (JA) biosynthesis. The *msd3* mutants had reduced levels of linolenic acid in both leaves and developing panicles that in turn decreased the levels of JA. Furthermore, the *msd3* panicle phenotype was reversed by treatment with methyl-JA (MeJA). Our characterization of *MSD1, MSD2,* and now *MSD3* demonstrates that JA-regulated processes are critical to the *msd* phenotype. The identification of the *MSD3* gene reveals a new target that could be manipulated to increase grain number per panicle in sorghum, and potentially other cereal crops, through the genomic editing of *MSD3* functional orthologs.

## 1. Introduction 

Grain number per panicle is a major determinant of grain yield in sorghum (*Sorghum bicolor* (L.) Moench) and other cereal crops [1,2,3,4,5,6]. Increased grain number and grain size, which are directly related to improved grain yield, are common goals during domestication of cereal crops, resulting in selection of genetic stocks with greater grain number and larger seeds [7]. Genetic, physiological, and agronomic studies performed in different environments have revealed that grain number per panicle is strongly correlated with grain yield per hectare [3,4,6,8,9]. Thus, understanding the mechanisms that govern the genetic determination of grain number per panicle and the effect of genetic manipulation of this trait have great potential to increase grain yield.

Currently, however, very little is known about how grain number per panicle is determined. Several features of the inflorescence contribute to the final grain number, including the number and size of the primary and secondary flower branches and fertility of spikelets (grass flowers). In sorghum, the inflorescence or panicle consists of a main rachis on which many primary branches are developed. Secondary branches and tertiary branches are developed from the primary branches [10,11]. The main inflorescence—primary, secondary, and tertiary branches—all end with a terminal triplet of spikelets, consisting of one sessile bisexual spikelet and two lateral pedicellate spikelets [12]. Below the terminal spikelets, one or more spikelet pairs can develop. These adjacent spikelet pairs consist of one sessile and one pedicellate spikelet.

In natural accessions of sorghum, only the sessile spikelets are fertile and capable of producing viable grains. The pedicellate spikelets occasionally develop anthers, but do not develop ovaries and eventually abort. Recently, we isolated and characterized a series of sorghum mutants in which both the sessile and pedicellate spikelets are fertile [11], and in which the number and size of the primary inflorescence branches are increased. These mutants were designated as multiseeded (*msd*) because their panicles are capable of producing more than 200% of the normal grain number per panicle relative to the nonmutated BTx623 [11]. Previously, we reported two *MSD* genes, *MSD1* and *MSD2,* which determine the fertility of the pedicellate spikelets. The *MSD1* gene encodes a TCP (teosinte branched/cycloidea/proliferating cell nuclear antigen)-domain plant-specific transcription factor that increases the expression of enzymes involved in the biosynthesis of jasmonic acid (JA) during panicle development [13]. The *MSD2* encodes a lipoxygenase (LOX) that catalyzes the first committed step of JA biosynthesis [14]. The elevated levels of JA in the wild type panicle may activate programmed cell death, leading to the arrest of the pedicellate spikelets in the wild type BTx623 [13,15]. Because the increase in JA is blocked in the *msd1* and *msd2* mutants, the pedicellate spikelets continue to develop into viable grains.

Here, we describe the identification of the *MSD3* gene, defined by a new *msd* mutant locus. *MSD3* encodes a major plastidial ω-3 fatty acid desaturase that catalyzes the desaturation of linoleic acid (18:2, 18 carbon chain with two double bonds) to linolenic acid (18:3, 18 carbon chain with three double bonds). The *msd3* mutants have dramatically reduced levels 18:3 fatty acid, as well as reduced levels of JA. JAs are lipid-derived cyclopentanone compounds that functionally resemble animal prostaglandins. Similar to the eicosanoid pathway of animals, JA is synthesized from linolenic acid (18:3) and hexadecatrienoic acid (16:3) through a series of steps of cyclization, reduction, and oxidation [16,17]. The reduced levels of 18:3 fatty acid in *msd3* mutants may lead to lower levels of endogenous JA. Indeed, the *msd3* phenotype can be reverted to the wild-type phenotype by application of MeJA. Our results indicate that the *msd* phenotype observed in the *msd3* mutants is caused by the reduction of endogenous levels of JA.

## 2. Results

### 2.1. Phenotype of the msd3 Mutants

**Figure 1:**
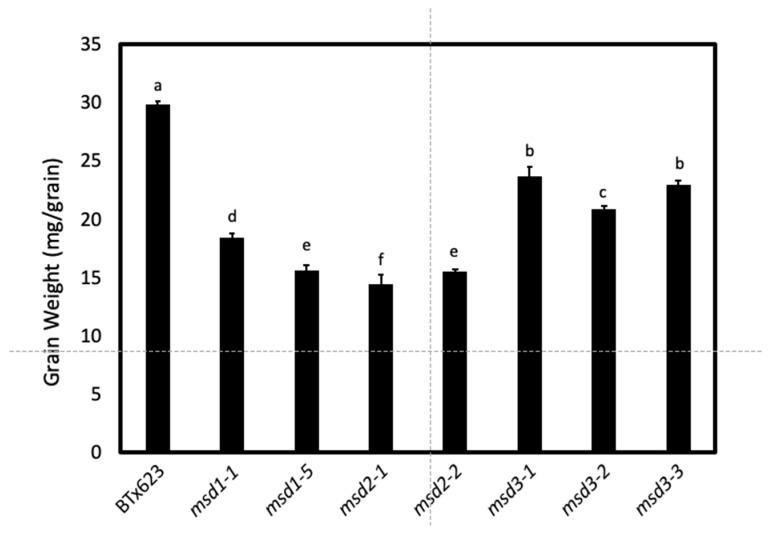
Comparison of grain weight (mg/grain) of *msd3* mutants vs. BTx623 and the *msd1* and *msd2* mutants. Grain weight was determined from plants planted on the Farm of Texas Tech University on Quaker Avenue in 2017. Four samples of 100 grains were weighed from each line. One-way ANOVA analysis revealed that grain weight varied significantly with an F-value of 276 and p-value of 1.8^−15^. Grain weights labeled with different letters are significantly different.

Here, we characterized a new group of multi-seeded (*msd*) mutants that include p6 (putative mutant #6), p14, p21, and p24. Similar to the *msd1* and *msd2* mutants, the *msd3* mutants also exhibit increases in number and size of the primary inflorescence branches (Figure S1) [11,13,14], and both their sessile and pedicellate spikelets are fertile. These coordinated phenotypic changes led to a ~2-fold increase in grain number per panicle. Complementation tests revealed that these *msd* mutants p6, p14, p21, and p24 represented a new locus that is distinct from the *msd1* and *msd2* loci (Table 1). These mutants were designated as *msd3*. Similar to the *msd1* and *msd2* mutants, the increase in grain number in *msd3* mutants was also associated with reduction in grain size [11,13,14]. However, the *msd3* mutants had larger grains than the *msd1* and *msd2* mutants, (Figure 1), making *msd3* potentially useful trait for improving grain yield and grain appeal.

### 2.2. Identification of the MSD3 Gene

**Figure 2:**
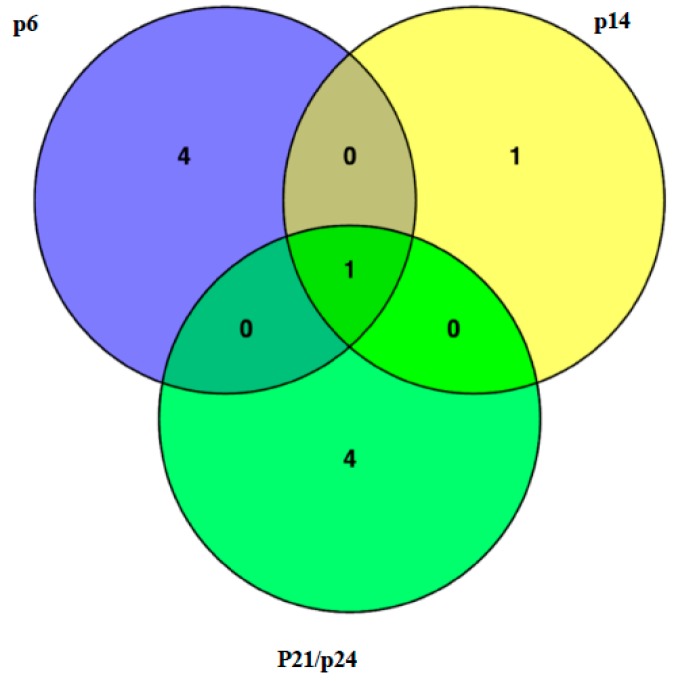
Identification of the *Msd3* gene by MutMap. Four F2 populations, BTx623*p6, BTx623*p14, BTx623*p21, and BTx623*p24, were subjected to sequencing of 20 bulked F2 mutants. The number in each circle represents the number of homozygous deleterious mutations. Only one gene, Sorbi_3001G407600, was commonly mutated in all four populations. In p21 and p24, the *MSD3* gene was mutated at the same position.

**Figure 3:**
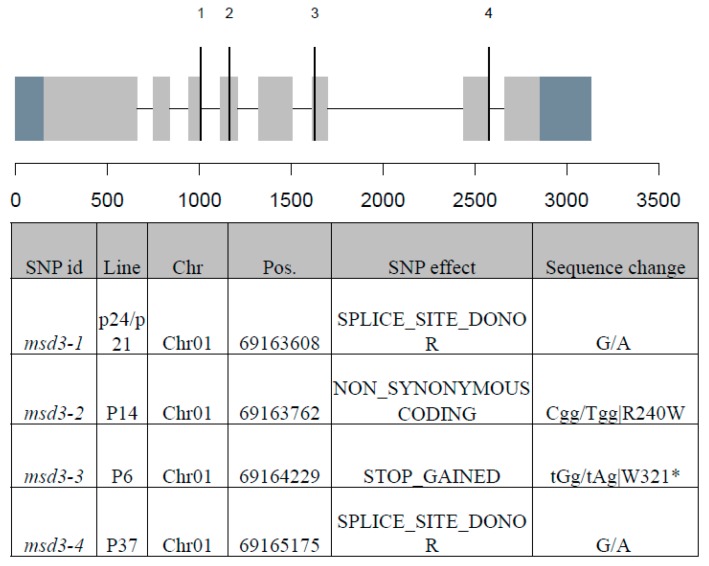
*MSD3* gene model and mutations. The *MSD3* gene, Sorbi_3001G407600, spanning a genomic sequence of 3132 bp, with a CDS of 1356 bp, encodes a protein of 451 amino acids. The gene has eight exons (grey boxes) and seven introns (black line). The vertical lines indicate mutation sites as described in the table above.

The *MSD3* gene was identified through bulk segregant analysis of whole-genome sequencing data with an in-house bioinformatics pipeline, as described in rice and sorghum [18,19]. Previously, we crossed p6, p14, p21, and p24 to BTx623 and derived four F_2_ populations. After bioinformatics analysis of the four bulked F_2_ pools, we identified only one gene (Sorbi_3001G407600) that carried homozygous mutations in all four bulked F_2_ pools (Figure 2). The genomic sequence of *MSD3* is 3132 bp in length, with a CDS of 1356 bp that encodes a protein of 451 amino acids. Two putative *msd* mutants, p21 and p24, harbored a mutation that converted the G residue at Chr01_69163608 to A, creating a splice site mutation at the junction of the third exon and the third intron (Figure 3). Both were renamed as *msd3-1*. The mutant p14, renamed *msd3-2*, harbored a G-to-A transition at Chr01_69163762 that created a nonsynonymous mutation, R240W, in the MSD3 protein. Mutant p6, renamed *msd3-3*, harbored a G-to-A transition at Chr01_69164229, resulting in a premature stop codon (W321*). Subsequently, we identified another mutation in the *MSD3* gene from the sequenced mutant library (ASR106, 25M2-1370) at Chr01_69165175, which created a splice site mutation at the junction of the seventh exon and the seventh intron [20]. The homozygous mutant (p37) exhibited the expected *msd* phenotype and was named as *msd3-4*.

**Figure 4:**
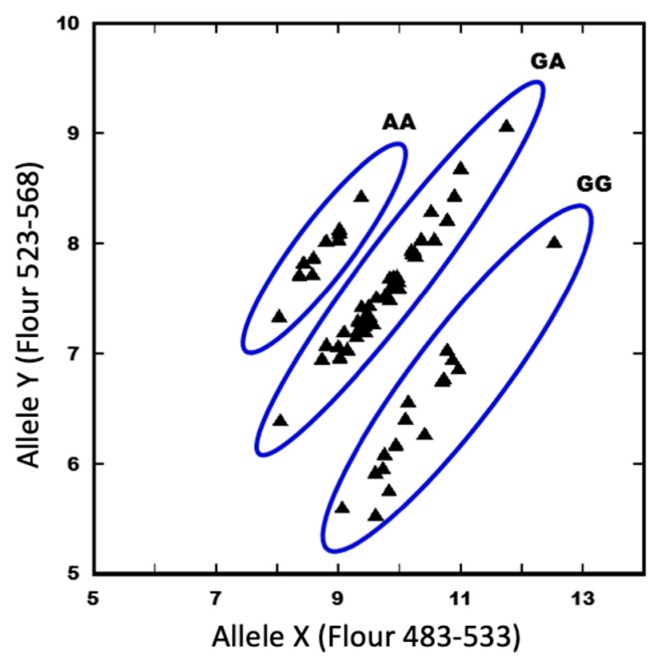
Genotyping of *msd3-4* F_2_ backcrossed population by KASP (Kompetitive Allele-Specific PCR) SNP (single nucleotide polymorphism) analysis. The x-axis represents the endpoint fluorescence data at 483–533 nm (allele X or wild type allele) and the y-axis represents the endpoint fluorescence data at 523–568 nm (allele Y or mutant allele). Genotype for individual F_2_ plants was determined by cluster analysis of the endpoint KASP assay. AA, homozygous *msd3-4*; GG, homozygous wild-type (WT); and GA, heterozygous WT.

To determine whether these mutations in the *MSD3* gene resulted in the *msd* phenotype, we made pairwise crosses among these four *msd3* mutants; all F_1_ plants from these crosses exhibited the *msd* phenotype (Table 1). By contrast, F_1_ plants resulting from crosses between *msd3* and *msd1*, *msd3* and *msd2*, or *msd2* and *msd1* exhibited the wild type panicle phenotype. Together, these results indicate that Sorbi_3001G407600 is the *MSD3* gene. Furthermore, we tested the co-segregation of the *msd3-4* mutation by Kompetitive allele-specific PCR (KASP) (Figure 4). Among 63 individual F_2_ plants derived from the cross of BTx623 * *msd3-4* (p37), 15 plants were scored as AA at the mutation site, and all 15 lines exhibited the expected *msd3* phenotype. Fifteen plants were scored as GG, and 33 were scored as GA; these two genotypic classes, GG (wild-type, WT) and GA (heterozygote), exhibited that the wild-type panicle structure indicating that the *msd3* mutation is recessive. These results also showed that the causal mutation in the *msd3-4* co-segregated perfectly with the *msd* phenotype.

### 2.3. MSD3 is FAD7, a Major Plastidial ω-3 Fatty Acid Desaturase

Sequencing analysis indicates that the *MSD3* gene (Sorbi_3001G407600) encodes a plastidial fatty acid desaturase that adds a double bond to the ω-3 carbon of linoleic acid (18:2) to convert it to linolenic acid (18:3). The sorghum genome has two plastid-targeted ω-3 fatty acid desaturases, Sorbi_3001G407600 and Sorbi_3002G430100. However, in many plants, *FAD7* is the major plastidial linoleic acid desaturase [21,22,23,24,25]. Based on the panicle phenotype of the *msd3* mutant, we reasoned that *MSD3* could be *FAD7* rather than *FAD8*. To determine whether *MSD3* is *FAD7* or *FAD8*, we constructed a matrix of identity between the two sorghum genes using the annotated *FAD7* and *FAD8* genes from rice and maize (Figure S2 and Table S1). *MSD3* exhibits 84.8% identity with rice *FAD7* (OsFAD7_LOC_Os03g18070.1) and 74.6% identity with rice *FAD8* (OsFAD8_LOC_Os07g49310.1), whereas the other linoleic acid desaturase (Sorbi_3002g430100) shows 76.2% identity with rice *FAD7* but 81.5% with rice *FAD8*. Therefore, we concluded that *MSD3* is *FAD7* not *FAD8*.

*FAD7* appears to be the major ω-3 fatty acid desaturase in sorghum. To determine the relative transcript abundance of the two plastidial ω-3 fatty acid desaturases in the developing panicle of the wild type BTx623, we compared the transcript levels of *FAD7* and *FAD8* using qPCR with three biological and three technical replicates. Comparing with the internal gene control EIF4α, the average cycle threshold (Ct) to detect *FAD8* transcript was 24.2 cycles, while the Ct number to detect *FAD7* transcript was 19.8. This difference in Ct translated to a relative abundance of *FAD7* transcript 21-fold over *FAD8* according to the calculation method as described previously [26]. This relative abundance was largely consistent with the trends of online data (Figure S3).

**Figure 5:**
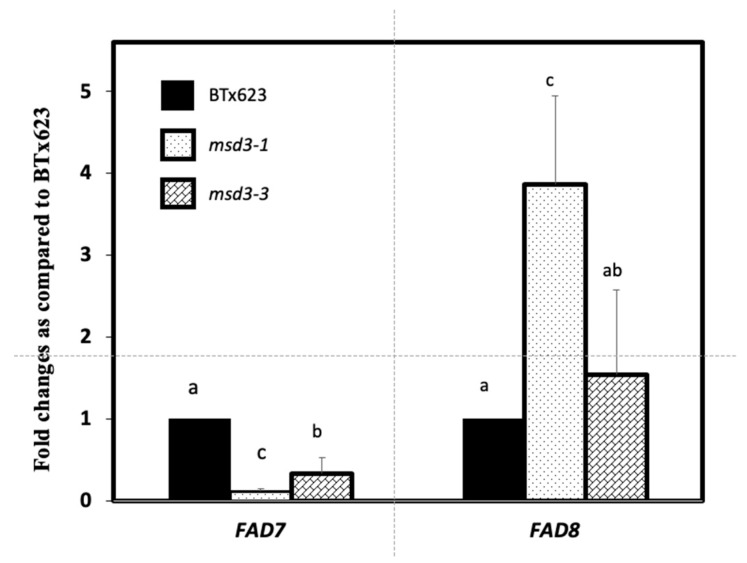
Expression of *FAD7* and *FAD8* genes in developing panicles. Relative change in *FAD7* and *FAD8* mRNA levels in *msd3* mutants in comparison to BTx623 (normalized as 1).

To determine which of two plastidial ω-3 fatty acid desaturases were affected in the *msd3* mutants, we compared the abundance of *FAD7* and *FAD8* transcripts in the *msd3-1* and *msd3-3* mutants relative to the wild type BTx623 (Figure 5). The abundance of *FAD7* was reduced to 10% in *msd3-1* and 30% in *msd3-3* in comparison to the wild type *FAD7* levels. Because the primers of *FAD7* and *FAD8* were designed based on the 3′-UTR sequences, which lie outside of the mutation sites, the low levels of the *FAD7* and *FAD8* transcripts may be due to truncated RNAs that do not encode active proteins. On the other hand, the transcript levels of *FAD8* were increased by 3.8-fold and 1.5-fold in the *msd3-1* and *msd3-3* mutants relative to the levels of BTx623, respectively, indicating that only the transcript abundance of the *FAD7* gene was significantly reduced in the *msd3* mutants. The slight increase in the abundance of *FAD8* gene suggested that the loss of function mutations in *FAD7* gene may be partially compensated by the elevated expression of *FAD8* (Figure 5). This result suggested that expression of *FAD7*, but not *FAD8*, was greatly reduced in the *msd3* mutants.

In addition to the two plastidial ω-3 fatty acid desaturases, the sorghum genome encodes two microsomal ω-3 fatty acid desaturases, Sorbi_3005G002800 and Sorbi_3008G003200, homologous to the Arabidopsis *FAD3* gene [25,27]. We searched our mutation database from the 256 sequenced mutant lines [20] and identified nonsynonymous mutations in all three genes (Table S2). However, none of the sequenced lines that harbored a mutation in one of the three ω-3 fatty acid desaturase genes exhibited a phenotype similar to the *msd* mutants.

### 2.4. Mutation in MSD3 Gene Dramatically Reduced the Levels of Linolenic Acid

**Figure 6:**
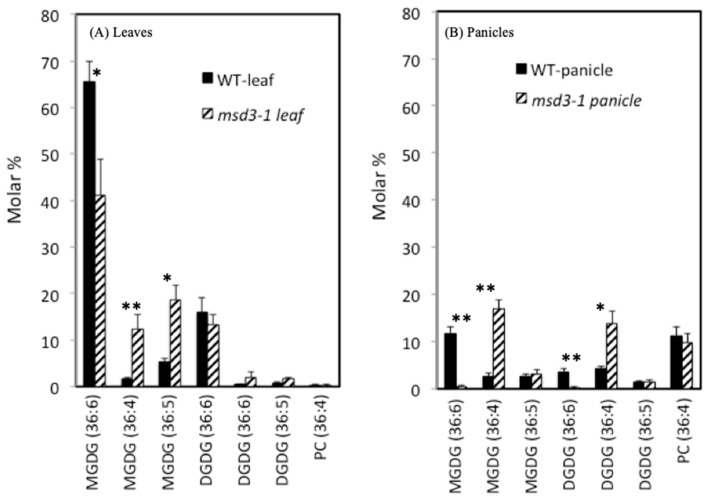
Lipid profiles of leaves and panicles from BTx623 and *msd3-3* (p6) mutants. Lipid profiles of the youngest mature leaves and panicles were carried out by the Lipidomics Center at Kansas State University. Numbers in parenthesis indicate the length and number of double bonds of the fatty acid moiety: (36:6) represents lipid species with two linolenic acids (18:3), (36:4) represents lipid species with two linoleic acids (18:2), and (36:5) represents lipid species with one linolenic acid and one linoleic acid moiety. Minor lipid species (molar percentage <1%) were not plotted, but can be found in Table S3. The difference between WT and *msd3-1* in each lipid species was analyzed by t-test. * *p* < 0.05; ** *p* < 0.01.

To understand how mutations in the *MSD3* (*FAD7*) gene lead to the dramatic change in panicle architecture and restore the fertility of the pedicellate spikelets, we first assessed the effect of the *msd3* mutations on lipid composition of leaves and panicles of the *msd3-1* mutant. In WT BTx623 leaves, 18-carbon fatty acids account for over 92% of total leaf lipids (Figure 6, Table S2). Levels of seven lipid species with molar percentage >1% are plotted in Figure 6. Galactolipids, including monogalactosyl diacylglycerol (MGDG) and digalactosyl diacylglycerol (DGDG), are the major lipids in leaves, accounting for over 90% of the total lipids. The lipid species with 36 carbon and six double bonds (36:6) consist of two linolenic acid molecules, whereas those with four double bonds (36:4) consist of two linoleic acid molecules, and species with five double bonds (36:5) consist of one linoleic acid and one linolenic acid. Sorghum has very low levels of 16-carbon fatty acids, and hexadecatrienoic acid (16:3) was not detectable in either leaves or panicles (Table S3).

As can be seen from Figure 6, mutation in *MSD3* decreased the levels of 36:6 lipid species and concomitantly increased the levels of 36:4 species. The ratio of linolenic acid to linoleic acid was 13.43 in the WT BTx623 leaves, falling to 2.48 in *msd3-3* leaves (Table S3). In general, panicles contained much less linolenic acid than in leaves. In WT BTx623 panicles, the ratio of linolenic acid to linoleic acid was 0.62, falling to 0.08 in the *msd3-3* mutant panicles. Thus, *msd3-3* panicles contained very little linolenic acid (7% vs. 38% in WT panicles). This result confirms that *MSD3* is an ω-3 fatty acid desaturase that catalyzes the desaturation of linoleic acid to linolenic acid in both leaves and panicles. Moreover, the dramatic effect of *msd3* mutations on linoleic acid desaturation was consistent with the idea that *MSD3* is sorghum *FAD7*, as *FAD7* has major effects on linoleic acid desaturation in other plant species [22,23,24].

### 2.5. The msd3 Phenotype Was Reverted by Treatment with MeJA

Because linolenic acid (18:3) is a substrate for JA biosynthesis [28], we next asked whether the *msd* phenotype of the *msd3* mutants was due to a deficiency of JA. For this purpose, we then measured the level of JA in developing panicles of *msd3-1* and BTx623. The JA level was reduced from 709 ng/g fresh weight (FW) in the wild type to 409 ng/g FW in *msd3-1* (Figure S4).

**Figure 7:**
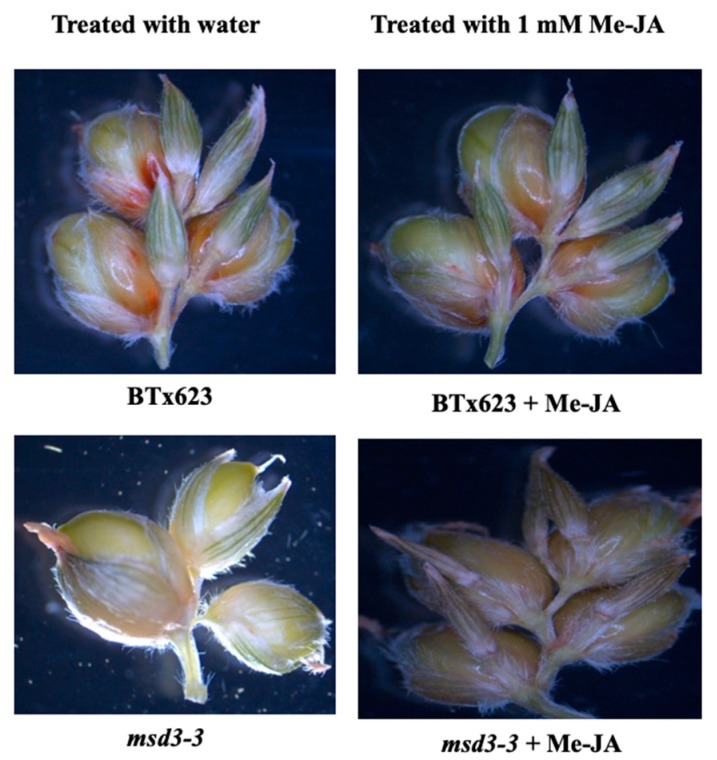
Reversion of the *msd* panicle phenotype by jasmonic acid (JA). 0.05% Tween-20 in water or 1 mM methyl-JA (MeJA) in 0.05% Tween-20 was directly applied to the central whorl of BTx623 and *msd3-3* (p6) plants every other day, starting from when the plants had eight fully expanded leaves and continuing until they had 10 fully expanded leaves. Shown in the figure were a few spikelets to demonstrate the reversion of the fertile pedicellate spikelets of the *msd3* mutant to sterile spikelets by the addition of MeJA. The effect of JA treatment on an entire primary inflorescence branch was shown in Figure S5.

To further demonstrate if the decrease in JA led to the *msd* phenotype, we applied MeJA to the whorls of BTx623 and the *msd3-1* mutant every other day, starting from when the plants had eight fully expanded leaves until the plants had 10 fully expanded leaves. Because the pedicellate spikelets never produce viable grains in BTx623 and other natural accessions, the reversion of the pedicellate spikelets from grain-bearing perfect flowers to sterile spikelets is a reliable indicator to demonstrate the effect of JA treatment. As shown in Figure 7, the pedicellate spikelets of the wild type BTx623 were sterile while that of *msd3-3* mutant were fertile and produced viable grain. After treatment with MeJA, the pedicellate spikelets of *msd3-3* mutants became sterile spikelets as in the wild type BTx623. Furthermore, the grain size of the *msd3* mutant also restored to similar size as the wild type BTx623 (Table S4). This result indicates that the lack of sufficient JA in the *msd3* panicle resulted in full fertility in the pedicellate spikelets. 

## 3. Discussion

In this study, we characterized a new group of *msd* mutants, *msd3*, that exhibits increases in the size and number of the primary branches and fertile pedicellate spikelets, similar to the panicle structure previously reported in the *msd1* and *msd2* mutants [13,14]. Using bulk segregant analysis of four independent F_2_ populations, we identified that all causal mutations are located within the *FAD7* (Sorbi_3001G407600) gene. Because *FAD7* is a major plastidial fatty acid desaturase, loss-of-function mutations in the *FAD7* gene resulted in a dramatic reduction in the content of linolenic acid, a substrate of JA, in both leaves and panicles. The reduction in JA levels in *msd3* panicles and the reversion of the *msd3* phenotype to the wild type by application of MeJA indicate that the *msd* phenotype in *msd3* mutants is probably due to insufficient levels of JA to activate the programmed cell death pathway leading to the arrest of the pedicellate spikelets. This conclusion is consistent with the previously reported mechanisms in *msd1* and *msd2* mutants [13,14].

*MSD3* (*FAD7*) appears to the predominant form of ω-3 fatty acid desaturase in sorghum. Most plants, including sorghum, have multiple forms of microsomal and plastidial ω-3 fatty acid desaturases that catalyze the formation of linolenic acids from linoleic acid. Arabidopsis has one microsomal fatty acid desaturase, originally known as *FAD3,* and two closely related plastidial ω-3 desaturases, *FAD7* and *FAD8* (plastidial) [21,22,23,24,29]. Mutations in all three genes, *FAD3, FAD7,* and *FAD8*, are required to reduce the linolenic acid levels sufficiently to affect JA-regulated processes such as male sterility and the defense response to insect attack [29,30,31]. However, loss-of-function mutation in *FAD7* alone blocks the JA-induced responses in tomato [22]. The sorghum genome encodes two microsomal ω-3 fatty acid desaturases, Sorbi_3005G002800 and Sorbi_3008G003200, which are homologous to Arabidopsis *FAD3* [25,27], and two plastidial fatty acid ω-3 desaturases, Sorbi_3001G407600 and Sorbi_3002g430100, which are homologous to Arabidopsis *FAD7* and *FAD8* [27,32]. Loss-of-function mutations in *MSD3* (*FAD7*) alone decreased the ratio of linolenic acid to linoleic acid from 13.43 to 2.48 in leaves, and from 0.62 to 0.08 in panicles, resulting in dramatic changes in panicle architecture, and restoration of the fertility of the pedicellate spikelets. As noted above, *MSD3* encodes one of the four ω-3 fatty acid desaturases, but the roles of the other three enzymes in fatty acid desaturation remains unclear. Based on their relative expression levels (Figure S3), these genes may not contribute much to the desaturation of linoleic acid except at specific developmental stages. Thus, *MSD3* (*FAD7*) plays a major role in the desaturation of linoleic acid to linolenic acid in sorghum.

Linolenic acid (18:3) and hexadecatrienoic acid (16:3) are the substrates for the lipid-derived JAs [16,17]. Because sorghum has non-detectable levels of hexadecatrienoic acid, linolenic acid serves as the main substrate for JA biosynthesis. We propose that the dramatic decrease in linolenic acid in the *msd3* mutants may result in deficiency of JA, which failed to arrest the development of the pedicellate spikelets in sorghum. Thus, we determined the JA levels in the developing panicles of BTx623 and *msd3-1* mutant. Although JA level in the *msd3-1* mutant panicle was significantly reduced to 409 ng/g FW from 709 ng/g fresh weight (FW) of the wild type, the *msd3* panicles still contained substantial levels of JA. Next, we tested if the *msd3* phenotype, such as fertile pedicellate spikelets, can be reversed by JA treatment. As shown in Figure 7 and Figure S5, the fertile pedicellate spikelets on the *msd3* panicle was reverted to sterile spikelets as in the BTx623. Thus, it is more likely that the dramatic reduction in linolenic acid in *msd3* mutants may lead to lower levels of JA, which failed to trigger the programmed cell death pathway and allowed the pedicellate spikelets to develop viable grains. This conclusion is also supported by our previous reports on *msd1* and *msd2* mutants [13,14].

The role of JA in the mediation of the *msd* phenotype in sorghum may be closely related to its role in maize tassel development by functioning at the level of sexual determination. Maize is monoecious with separate staminate (male) and pistillate (female) inflorescences called the tassel and the ear, respectively. During early floral development, the florets in both the tassel and the ear spikelets are bisexual. The monoecious nature is conferred by the selective abortion of pistillate organs in tassel florets [15]. A large number of sex-determination mutants (tasselseed) have been identified in maize [33]. The *TS1* encodes a lipoxygenase that catalyze the conversion of free linolenic acid to 13(S)-hydroperoxylinolenic acid, the first committed step in JA biosynthesis. The sorghum *MSD2* gene is an ortholog of the maize *TS2* gene [14]. The maize *TS2* gene encodes a short-chain alcohol dehydrogenase, probably involved in JA biosynthesis [34]. Another tasselseed mutant was created by knocking out the duplicated orthologs of OPR3, a major OPR (12-oxo-phytodienoic acid reductase) gene in Arabidopsis that acts in JA biosynthesis [35]. The resulting maize *opr7opr8* double mutant is phenotypically similar to *ts1* and *ts2* mutants and can be reverted to normal tassel phenotype by treatment with MeJA [36]. The maize *TS5* gene encodes a wound-inducible gene, CY94B, which serves as an enzyme that inactivates the biologically active JA-isoleucine [33]. Although the causal mutation in the *TS5* gene has not been identified, the mutation of *ts5* is dominant and leads to the overexpression of CY94B, decreased JA-isoleucine, and a concomitant increase in the deactivated 12OH-JA-isoleucine. As with the maize *ts1* and *ts2* mutants, the tasselseed phenotype was reverted by JA treatment. It is well-established that JA plays a critical role in the maintenance of the staminate state of the tassel by suppressing the development of female flower organs through JA-induced programmed cell death [15].

Sorghum is hermaphroditic and produces perfect flowers with both male and female floral organs. Like many other grasses, sorghum produces two types of spikelets depending on the mode of attachment to the inflorescence axis. The sessile spikelets, directly attached to the inflorescence axis, contain perfect flowers and produce viable grains. The pedicellate spikelets, attached to the inflorescence axis through a short petiole called pedicel, occasionally produce anthers but never produce mature ovaries, and thus, become sterile spikelets in mature panicles. In sorghum, *msd* mutants produce both sessile and pedicellate spikelets that contain perfect flowers and can develop viable grains. Sorghum may employ similar mechanisms to control the fertility of the pedicellate spikelets as reported in maize in controlling the ovary development in tassels. In the wild type BTx623, both sessile and pedicellate spikelets have both male and female floral organs initially but the floral organs, especially the ovary, in the pedicellate spikelets become arrested in a late development stage [13]. The abortion of the ovary has been shown to be regulated by JA-induced programmed cell death as in maize tassels [13,15]. As previously reported, the *MSD1* gene encodes a TCP-transcription factor that activates many genes involved in JA biosynthesis and *MSD2* encodes a lipoxygenase that catalyzes the first committed step of JA biosynthesis from linolenic acid [13,14]. The discovery of the *MSD3* gene as a predominant form of ω-3 fatty acid desaturase responsible for the conversion of linoleic acid to linolenic acid highlights the importance of the JA regulatory module(s) in the control of the fertility of the pedicellate spikelets in sorghum.

The mechanisms determining the staminate state of tassels in maize and that suppressing the development of the pedicellate spikelets in sorghum may also differ. All maize tasselseed mutants are male sterile due to the ectopic growth of the silks. However, the sorghum *msd* mutants develop both anthers and ovaries. Male sterility was never observed in the sorghum *msd* mutants. In addition, JA may also contribute to panicle development in sorghum, because all *msd* mutants exhibit increases in the number and size of the primary inflorescence branches [11,13,14].

## 4. Materials and Methods

### 4.1. Plant Materials

Sorghum (*Sorghum bicolor* (L.) Moench) *msd* mutants were identified from a pedigreed sorghum mutant library that was developed by mutagenizing the sorghum inbred line BTx623 seeds with the chemical mutagen ethyl methane sulfonate (EMS) [37]. The wild type BTx623, the *msd* mutants, and their backcrossed F_2_ populations were planted in the Agricultural Experiment Station at Lubbock, TX (33′39” N, 101′49” W) of the Agricultural Research Service of the United States Department of Agriculture (USDA-ARS) in May 2017. During the late grain-filling stage, when the *msd* phenotype could be easily observed, leaf samples were collected from each of the confirmed homozygous *msd* mutants to prepare genomic DNA as described previously [38].

The *msd* mutants isolated from the library harbored a high density of EMS-induced mutations [20]. To decrease the number of background mutations, these mutants were backcrossed (BC) to WT BTx623 for three generations before use in this study. Moreover, the new *msd* mutants were also crossed to the known *msd1* and *msd2* mutants, as well as to each other, to determine whether they represented alleles of known *msd* loci or new locus.

### 4.2. Gene Identification by Next-Generation Sequencing

Homozygous *msd* mutants were identified from the backcrossed F_2_ populations of p6, p14, p21, and p24 during the grain-filling stage, during which the *msd* phenotype could be easily scored. Genomic DNA pooled from the homozygous *msd* mutants from each F_2_ population was sequenced to >20× coverage on an Illumina HiSeq 2000 through a service from BGI (https://www.bgi.com/us/). Causal mutations in these populations were identified by an inhouse bioinformatic pipeline described previously [19]. Briefly, low-quality reads, adapter sequences, and contamination were excluded from the raw reads, and then the clean reads were aligned to the sorghum reference genome v3.0 with Bowtie2 [39]. SNP (single nucleotide polymorphism) calling was performed by Samtools and Bcftools using only reads with mapping and sequencing quality greater than Q20 [40]. The read depth for true SNPs was set from 3 to 50. Because EMS induces only G/C-to-A/T transition mutations [41], only homozygous G/C to A/T SNPs were used for prediction of effects on gene function by the Ensembl Variant Effect Predictor [42]. Homology analysis and functional annotation of candidate genes were obtained from Gramene database release 39 [43] (http://www.gramene.org).

### 4.3. Confirmation of the Causal Mutation with KASP

The BC_3_F_2_ population consisting of 63 F_2_ offspring was utilized for this study. Based on putative SNPs discovered through the sequencing analysis described above, three types of SNP–KASP primers were designed [44,45] and synthesized by Integrated DNA Technologies (Coralville, IA, USA). Genotyping analyses were performed using extracted genomic DNA from leaf tissues of sorghum plants collected from the field. SNP genotype in each offspring was determined by Kompetitive Allele Specific PCR (KASP) chemistry (www.lgcgenomics.com) with some modifications [44]. Analysis of genotypes was conducted at the Plant Stress and Germplasm Development Unit at Lubbock, TX, USA. Phenotype data for the population were gathered at the full maturity stage of the panicle. Co-segregation analyses were conducted based on the correspondence of genotype to phenotype and chi-square test.

### 4.4. Lipidomic Assay

Samples were obtained from ~5 cm^2^ from the middle lamina of the first mature leaf (the youngest leaf with a visible leaf collar) and from developing panicles (approximately 3 cm long). The samples were quickly immersed in liquid nitrogen and stored at −80 °C until use. Lipids were extracted and analyzed following the procedure established at the Lipidomic Center of Kansas State University (https://www.k-state.edu/lipid/). The original protocol was modified as follows: instead of cutting leaves into small pieces, the leaf tissue was completely ground into a fine powder with liquid nitrogen chilled mortars and pestles. The plant material was then treated with hot isopropanol and 0.01% butylated hydroxytoluene (preheated to 75 °C). The resultant lipid extract was dissolved in chloroform and stored at −80 °C. Lipid extracts were dried with a stream of nitrogen gas, packed in dry ice, and shipped to the Lipidomic Center of Kansas State University for lipid analysis. The tissue used for extraction was dried in an oven at 65 °C for 1 week to estimate the total dry weight of tissue used for extraction of each lipid sample.

### 4.5. Jasmonic Acid Determination

Panicles at stage 4, when the fate of the pedicellate spikelets is determined [13,14], were collected and immediately frozen in liquid nitrogen. Five biological replicates were solvent-extracted, methylated, collected on a polymeric adsorbent using vapor-phase extraction, and analyzed using GC/isobutene chemical ion mass spectrometry (GC/CI-MS) [46]. For metabolite quantification, d_5_JA (Sigma–Aldrich, St. Louis, MO, USA) was used as an internal standard. The JA level in each sample was normalized to the mass of the panicle, and expressed as ng/g fresh weight (FW).

### 4.6. Quantitative Gene Expression Analysis of FAD7 and FAD8

Sorghum *msd3* and BTx623 plants were grown in a greenhouse with temperatures maintained at 28 °C (12 h day)/25 °C (12 h night) cycle. Panicle samples at stage 4, about 3 cm long, were collected, flash frozen in liquid nitrogen, and stored at −80 °C until use. Total RNA was extracted from a single panicle with Trizol reagent (Thermo–Fisher Scientific, Waltham, MA, USA), followed by column purification using the RNA Extraction kit from Sigma–Aldrich (Sigma–Aldrich, St. Louis, MO, USA). After purification, genomic DNA contaminants were removed by On-Column DNase I Digestion supplied in the Sigma kit (Sigma, St Louis, MO, USA). RNA concentration was measured using a BioSpectrophotometer^®^ Kinetic (Eppendorf, Hamburg, Germany). RNA quality was checked by electrophoresis on a 1% agarose gel. First-strand cDNA was synthesized from 1 µg RNA per sample using the Superscript 2 Reverse Transcriptase kit (Invitrogen, Carlsbad, CA, USA). The reaction mixture was incubated at 42 °C for 50 min followed by heat inactivation at 70 °C for 15 min. The resultant cDNA is used as a template for quantitative analysis of *FAD7* and *FAD8* genes with control eukaryotic translation initiation factor.

Real-time polymerase chain reaction (PCR) analysis was used to determine which plastidial fatty acid desaturase was affected in the *msd3* mutants. The Primer3 software was used to design primers for the *FAD7* and *FAD8* genes from sorghum. Combinations of forward and reverse primers with Tm above 60 °C were selected for real-time PCR analysis, and the annealing temperature was set to 53 °C. Because of the high sequence similarity among fatty acid desaturase genes, primers were designed within the 3′-UTR region to prevent non-specific binding to genes in the same family. 3′-UTR regions unique to *FAD7* (*FAD7* forward primer: 5′ TCC CTC AAA TCC CAC ATT 3‘, *FAD7* reverse primer: 5′ GAA GAG CAC CCG ACT TCT TT 3‘) and *FAD8* (*FAD8* forward primer: 5′ TGC ATG GAG GTT CAT ATA CTG C 3‘, *FAD8* reverse primer: 5′ AAT TCT GTT CTG TTT GGT TGG TG 3‘) were used to design primer pairs that amplified a product of ~100 base pairs. Internal control primers were designed against the gene encoding eukaryotic translation initiation factor 4 α (*EIF4α*) (SB_EIF4α Forward: 5′ CAA CTT TGT CAC CCG CGA TGA 3′ SB_EIF4αReverse: 5′ TCC AGA AAC CTT AGC AGC CCA 3′).

The cDNA was diluted five times with nuclease-free water, mixed with Roche Fast Start SYBR Green^®^ (Roche, San Francisco, CA, USA) along with specific primers and run on a Roche LightCycler 96. Three biological replicates (control and mutant samples) were used for gene expression analysis. The melt curve from the Light Cycler was analyzed by exporting the Δ*C*t and ΔΔ*C*t values to an Excel file. *EIF4*α was used as an internal control for analyzing the relative expression level differences of *FAD7* and *FAD8* genes.

### 4.7. Reversion of the msd3 Panicle Structure by MeJA

Treatment with MeJA was performed as described [13,14]. Briefly, 1 mL of 1 mM MeJA in 0.05% Tween-20 in water was applied to the central whorl every other day, starting when the plants had eight fully expanded leaves until the plants had 10 fully expanded leaves. Controls were treated with 1 mL of 0.05% Tween-20.

### 4.8. Statistical Analyses

Statistical analyses, such as one-way ANOVA and t-test, were run in Excel with the XRealStats add-in (http://www.real-statistics.com). Option ‘Tukey’s HSD’ was used for multiple comparisons.

## 5. Conclusions

We identified *MSD3* as *FAD7* (Sorbi_3001G407600), a major plastid-targeted ω-3 fatty acid desaturase. Loss-of-function mutations in *MSD3* (as in *msd3-1*, *msd3-3*, and *msd3-4*) result in low levels of linolenic acid, the substrate for JA biosynthesis. The *msd* panicle architecture was restored to the wild-type panicle phenotype by treatment with MeJA. The discovery that *MSD3* is *FAD7* provides further support for the idea that JA-regulated modules are responsible for suppressing the development of pedicellate spikelets in sorghum, as described previously in our characterization of the *msd1* and *msd2* mutants. Compared with *msd1* and *msd2* mutants, *msd3* mutants have higher grain weight indicating that *msd3* mutants may be better adapted to increasing sink capacity, and therefore may have better potential to increase grain yield. These findings of this study provide a new approach for genetic manipulation of grain numbers per panicle with the goal of increasing grain yield.

## Figures and Tables

**Table 1:** Complementation tests between *msd3*, *msd2*, and *msd1* mutants. The *msd3* mutants were crossed to each other and to *msd1-1* and *msd2-1* mutants by hand emasculation and manual pollination. The panicle phenotypes of the F_1_ plants were evaluated during the grain filling stage.

Mutant	*msd1-1 (p12)*	*msd2-1 (p4)*	*msd3-1 (p24)*	*msd3-2 (p14)*	*msd3-3 (p6)*
*msd1-1 (p12)*					
*msd2-1 (p4)*	WT				
*msd3-1 (p24)*	WT	WT			
*msd3-2 (p14)*	WT	WT	*msd*		
*msd3-3 (p6)*	WT	WT	*msd*	*msd*	
*msd3-4 (p37)*	WT	WT	*msd*	*msd*	*msd*

## Supplementary Materials

Supplementary materials can be found at https://www.mdpi.com/1422-0067/20/21/5359/s1.
