# Supplementary material for: Sorghum MSD3 Encodes an ω-3 Fatty Acid Desaturase that Increases Grain Number by Reducing Jasmonic Acid Levels
Source: Int J Mol Sci. 2019 Oct 28;20(21):5359. doi: 10.3390/ijms20215359 (PMC6862555; doi:10.3390/ijms20215359)
Supplement: Supplementary file 1 [file ijms-20-05359-s001.zip › ijms-606512-SI-final/ijms-606512-supplementary figures-proofdone.pdf]

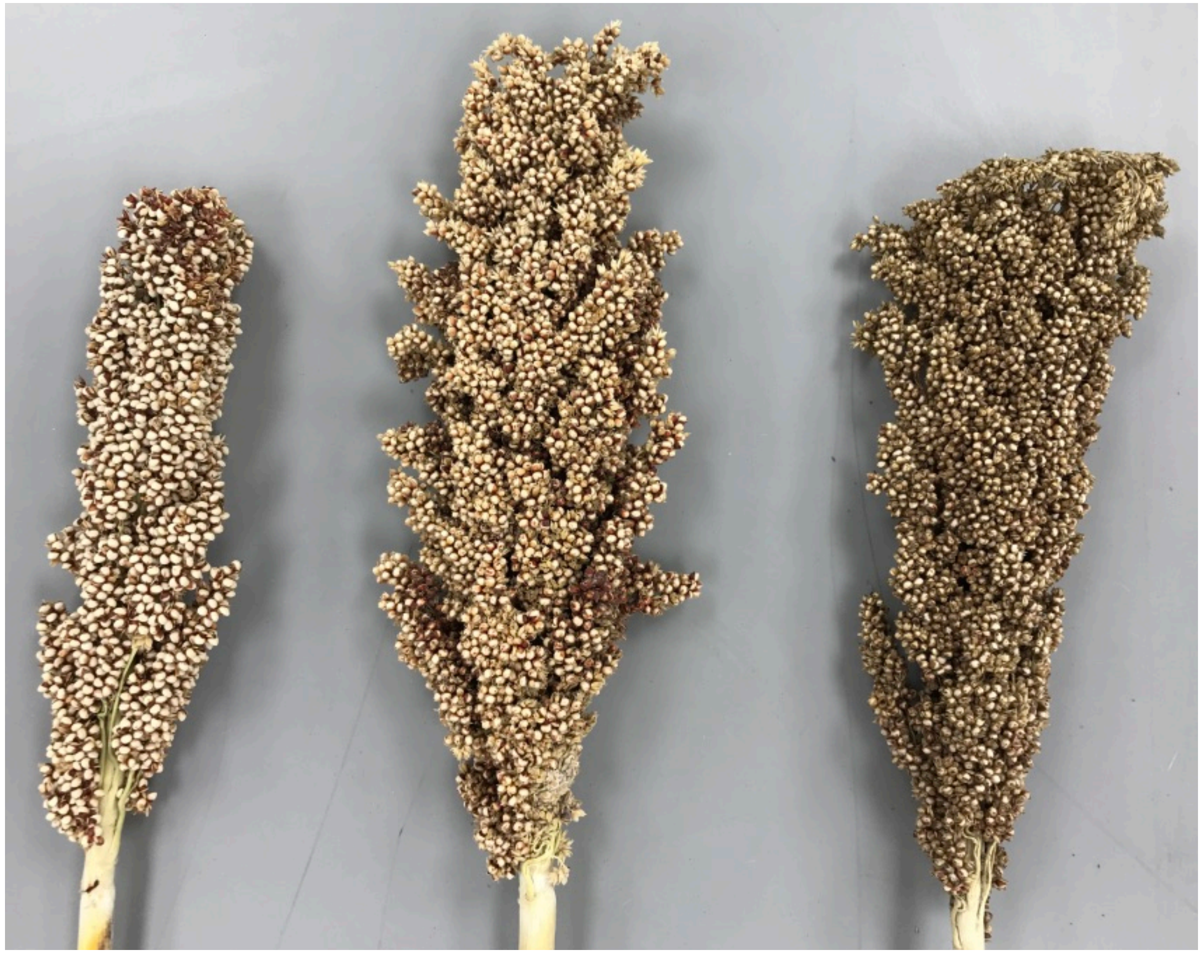

BTx623 *msd3-1* *msd3-3*

Supplementary Figure 1S. Panicle architecture of *msd3* mutants. Typical panicles of BTx623, *msd3-1,* and *msd3-3* were cut from the field from growing season of 2019 and photographed in the laboratory. The *msd3-2* and *msd3-4* were not planted in the field.

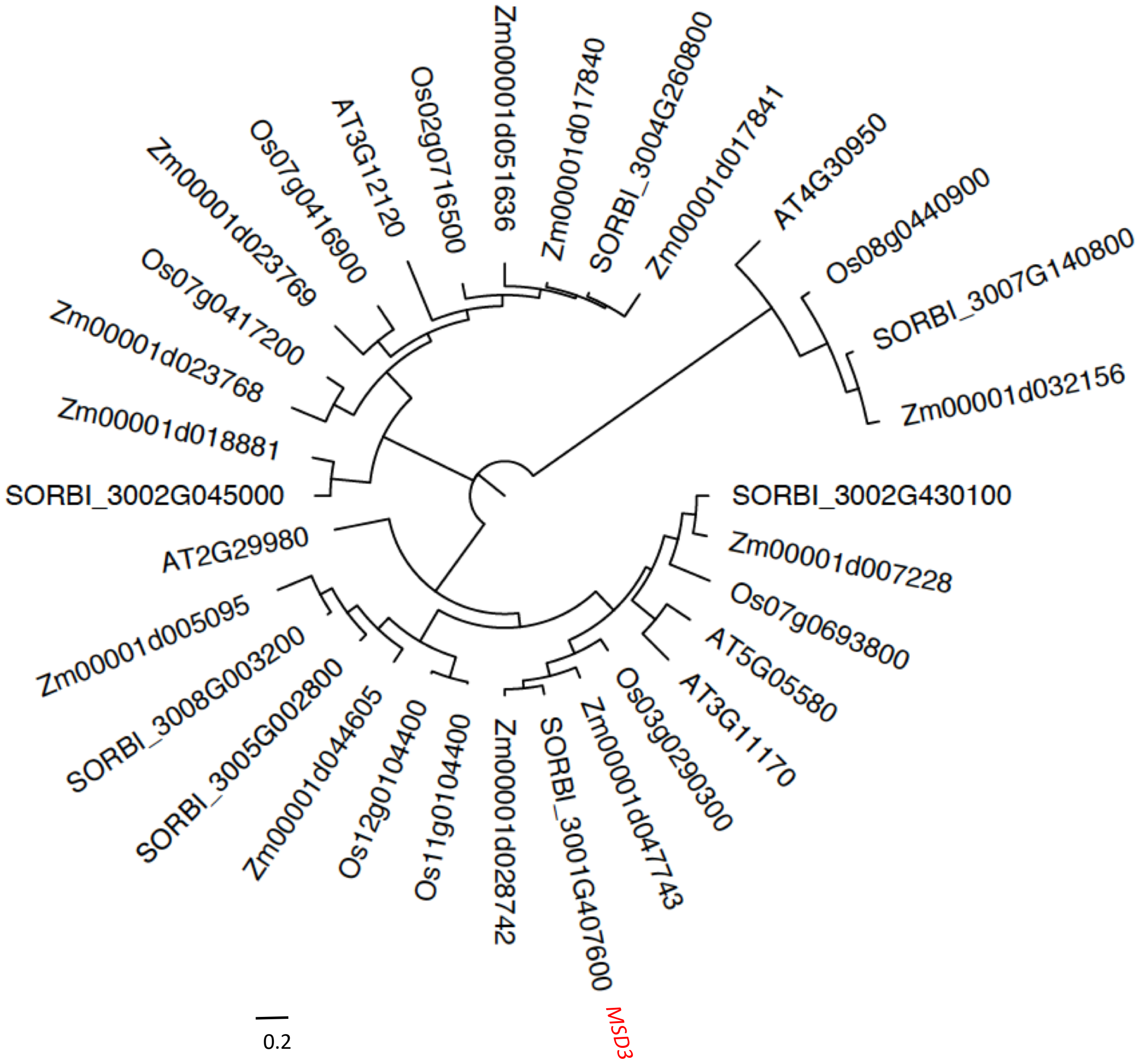

Supplementary Figure 2S: Phylogenic analysis of ω-3 fatty acid desaturases.

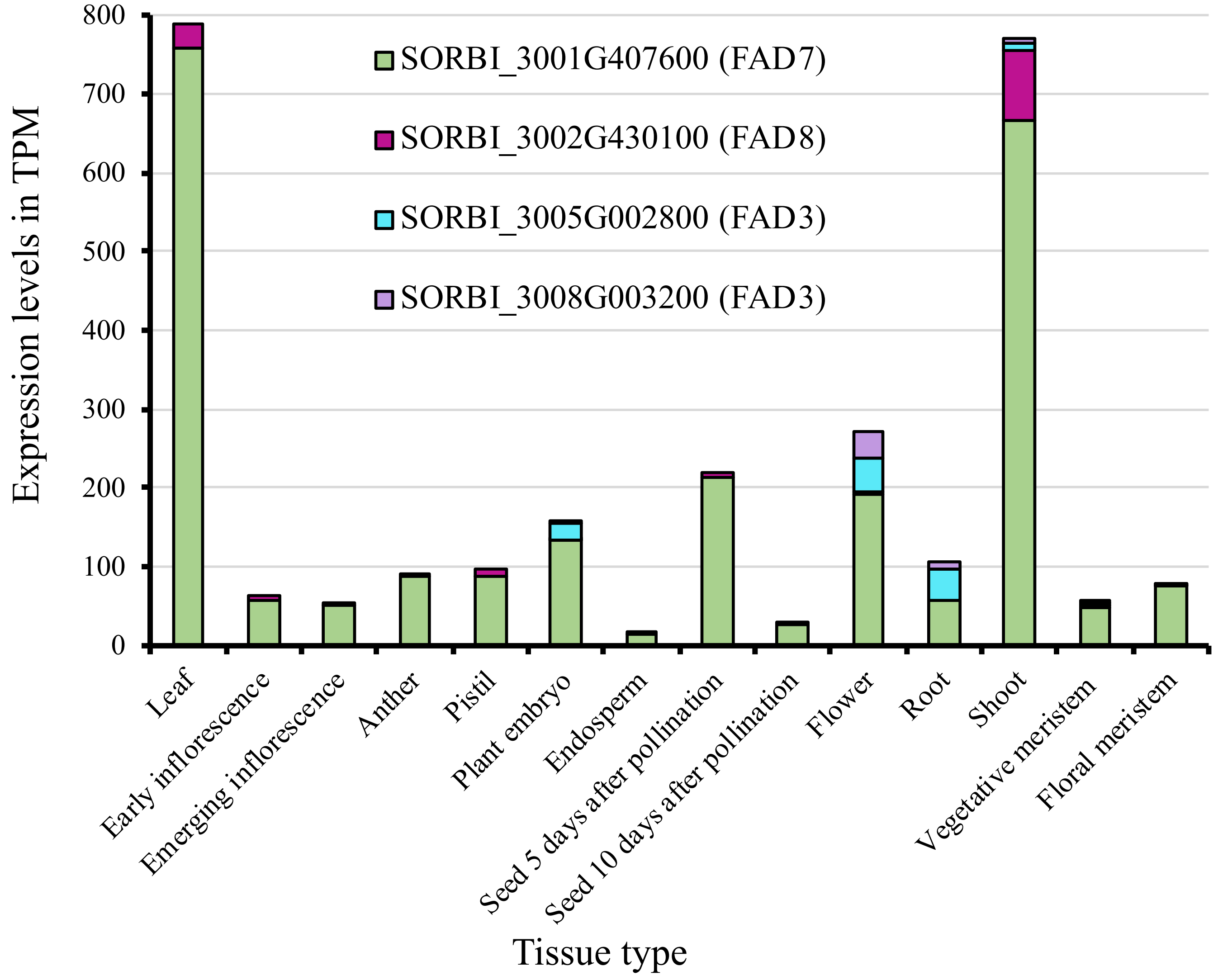

Supplementary Figure 3S: Tissue specific expression of sorghum ω-3 fatty acid desaturases. Tissue-specific expression of sorghum ω-3 fatty acid was annotated from an online data base (https://www.ebi.ac.uk/gxa/home).

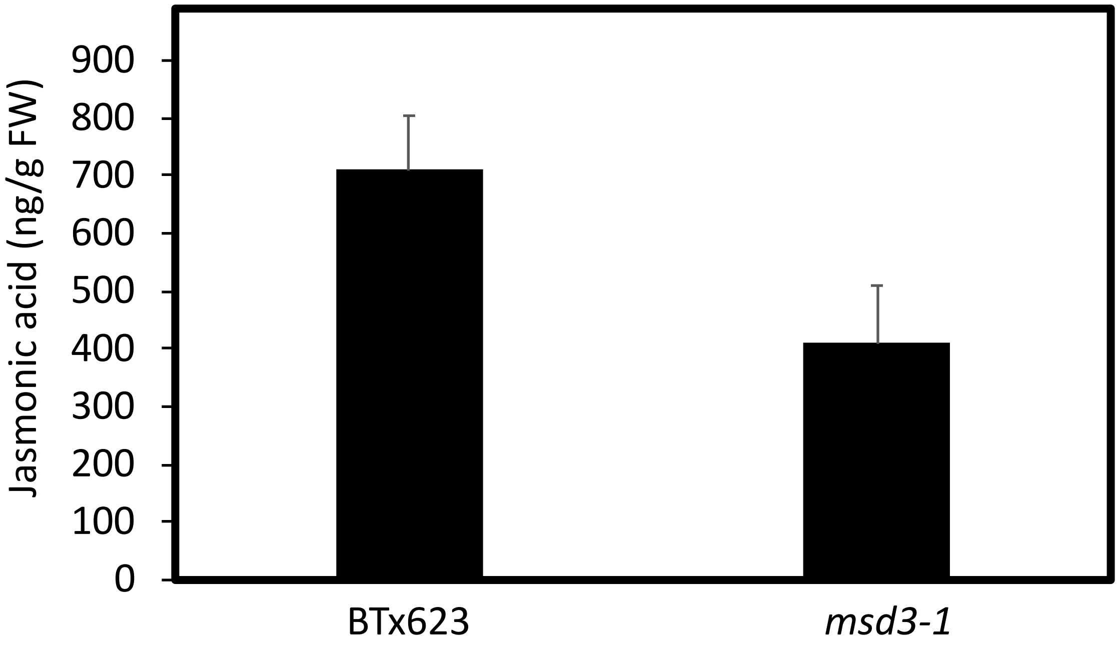

Supplementary Figure 4S. JA in developing panicles of BTx623 and *msd3-1* mutant.
Panicles at stage 4 (about 1.5 cm in length) were sampled and froze in liquid nitrogen. The content of cis-JA was expressed as ng/g fresh weight. T-test indicated the difference is significant with p-value=0.004.

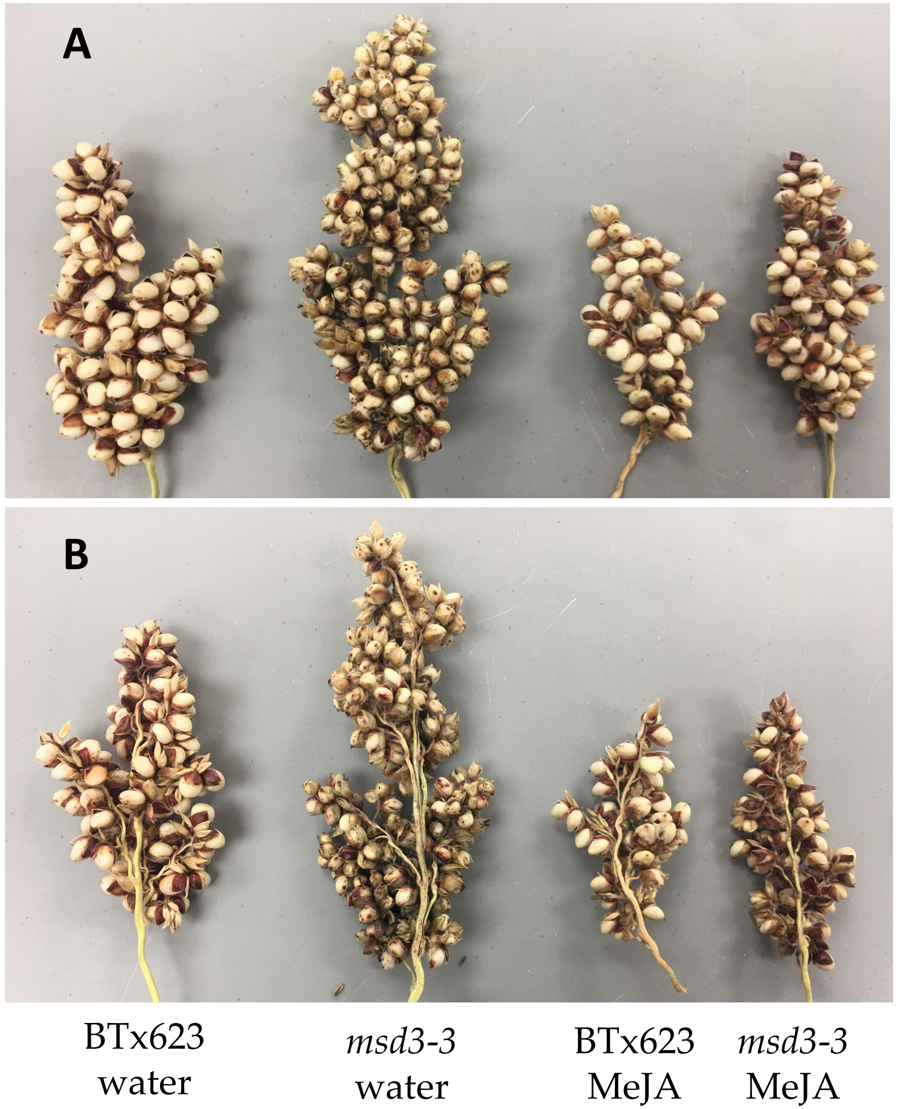

Figure 5S Primary Inflorescence Branches of BTx623 and *msd3-3* treated with 0.05% Tween-20 in water and 0.05% Tween-20 containing 1 mM MeJA in water. A typical primary inflorescence branch from the middle of the panicle were photographed. A. The view from outside of a primary inflorescence branch showing the increased grain number of the *msd3-3* mutant over the BTx623. B. The view from inside of a primary inflorescence branch showing the sterile pedicellate spikelets in BTx623, fertile pedicellate spikelets in msd3-3, reversion to sterile pedicellate spikelets after treatment with MeJA.
