# Supplementary material for: Sorghum MSD3 Encodes an ω-3 Fatty Acid Desaturase that Increases Grain Number by Reducing Jasmonic Acid Levels
Source: Int J Mol Sci. 2019 Oct 28;20(21):5359. doi: 10.3390/ijms20215359 (PMC6862555; doi:10.3390/ijms20215359)
Supplement: Supplementary file 1 [file ijms-20-05359-s001.zip › ijms-606512-SI-final/ijms-606512-SupplementaryTables-proofdone.docx]

**Supplementary Information.**

**Table 1.** *FAD7*/*FAD8* Identity Matrix.

|  | **OsFAD7_LOC_Os03g18070.1** | ***ZmFAD7*_Zm00001d047743_P001**  **Correct** | **MSD3** | ***ZmFAD8*_Zm00001d028742_P001**  **Likely *FAD7*** | **OsFAD8_LOC_Os07g49310.1** | **Zm00001d007228_T001Likely *FAD8*** | **Sobic.002G430100.1** |
| --- | --- | --- | --- | --- | --- | --- | --- |
| *OsFAD7*_LOC_Os03g18070.1 | 100 | 84.09 | 84.82 | 84.75 | 74.76 | 74.14 | 76.17 |
| *Zm****FAD7***_Zm00001d047743_P001 | 84.09 | 100 | 92.74 | 92.95 | 74.94 | 74.2 | 75.98 |
| MSD3 | 84.82 | 92.74 | 100 | 96.18 | 74.63 | 74.88 | 76.66 |
| *Zm****FAD8***_Zm00001d028742_P001 | 84.75 | 92.95 | 96.18 | 100 | 76.04 | 75.12 | 76.85 |
| *OsFAD8*_LOC_Os07g49310.1 | 74.76 | 74.94 | 74.63 | 76.04 | 100 | 79 | 81.55 |
| Zm00001d007228_T001 ***FAD8*** | 74.14 | 74.2 | 74.88 | 75.12 | 79 | 100 | 90.45 |
| Sobic.002G430100.1 | 76.17 | 75.98 | 76.66 | 76.85 | 81.55 | 90.45 | 100 |

Amino acid identity between *MSD3* and the *FAD7* or *FAD8* genes from rice and maize. *MSD3* has higher identity to rice *FAD7* than to rice *FAD8*. Because the maize gene *Zm****FAD8***_Zm00001d028742_P001 has 93% identity to *Zm****FAD7***_Zm00001d047743_P001, but 75% identity with Zm**FAD7**_Zm00001d047743_P001, it is likely the gene *Zm****FAD8***_Zm00001d028742_P001 is another copy of *FAD7*, rather than *FAD8* as annotated. Based on this assumption, *MSD3* has over 90% identity to both copies of *FAD7* and 75% identity to the presumed maize *FAD8*. Thus, *MSD3* is most likely *FAD7*.

**Table 2.** Mutations in Other ω-3 fatty acid desaturases.

| **Gene_ID** | **Gene Name** | **Sequenced Line** | **Mutation** |
| --- | --- | --- | --- |
| Sorbi_3005G002800 | *FAD2* | ARS249 | L203F |
| Sorbi_3008G003200 | *FAD2* | ARS137 | R401H |
|  |  | ARS16 | G138R |
|  |  | ARS106 | G61S |
| Sorbi_3002g430100 | *FAD8* | ARS146 | R33C |
|  |  | ARS23 | V39M |

The other three ω-3 fatty acid desaturases were searched for mutations from the sequenced mutant lines [20]. Although every gene has nonsynonymous mutation(s), none of these lines that harbor the mutation had *msd* phenotype.

**Table 3.** Mole percentage of major lipid species in the leaves and panicles of BTx623 and *msd3-3* (SBp6).

| **Lipid species** | **WT leaves**  **(mole% total lipid)** | | **WT panicles**  **(mole % total lipid)** | | **P6 leaves**  **(mole% total lipid)** | | **P6 panicles**  **(mole% total lipid)** | |
| --- | --- | --- | --- | --- | --- | --- | --- | --- |
|  | **ave** | **stdev** | **ave** | **stdev** | **ave** | **stdev** | **ave** | **stdev** |
| PC(36:4) | 0.38 | 0.13 | 11.34 | 1.76 | 0.38 | 0.11 | 9.81 | 1.95 |
| PC(36:3) | 0.10 | 0.06 | 5.22 | 0.24 | 0.02 | 0.05 | 3.94 | 1.01 |
| DGDG(36:4) | 0.48 | 0.12 | 4.35 | 0.43 | 2.02 | 1.34 | 13.92 | 2.68 |
| PE(36:4) | 0.12 | 0.04 | 2.87 | 0.22 | 0.14 | 0.05 | 2.92 | 0.65 |
| DGDG(36:3) | 0.27 | 0.13 | 2.80 | 0.31 | 0.32 | 0.12 | 3.42 | 0.77 |
| MGDG(36:4) | 1.81 | 0.21 | 2.60 | 0.70 | 12.43 | 3.17 | 16.90 | 1.94 |
| MGDG(36:3) | 0.12 | 0.04 | 0.85 | 0.48 | 0.23 | 0.15 | 1.75 | 0.56 |
| PE(36:3) | 0.01 | 0.01 | 0.59 | 0.19 | 0.01 | 0.01 | 0.36 | 0.07 |
| PI(36:3) | 0.01 | 0.01 | 0.58 | 0.07 | 0.01 | 0.01 | 0.48 | 0.08 |
| PG(36:4) | 0.01 | 0.01 | 0.15 | 0.03 | 0.01 | 0.00 | 0.14 | 0.03 |
| PG(36:3) | 0.00 | 0.00 | 0.13 | 0.09 | 0.00 | 0.00 | 0.07 | 0.02 |
| PA(36:3) | 0.00 | 0.00 | 0.08 | 0.03 | 0.00 | 0.00 | 0.09 | 0.03 |
| PS(36:3) | 0.00 | 0.00 | 0.02 | 0.00 | 0.00 | 0.00 | 0.00 | 0.00 |
| PS(36:4) | 0.00 | 0.00 | 0.01 | 0.00 | 0.00 | 0.00 | 0.02 | 0.01 |
| PI(36:4) | 0.03 | 0.01 | 0.86 | 0.12 | 0.05 | 0.03 | 0.85 | 0.16 |
| MGDG(36:5) | 5.28 | 0.81 | 2.66 | 0.46 | 18.72 | 3.21 | 3.03 | 1.10 |
| PC(36:5) | 0.34 | 0.09 | 2.42 | 1.76 | 0.18 | 0.17 | 1.24 | 1.25 |
| DGDG(36:5) | 0.73 | 0.19 | 1.50 | 0.27 | 3.24 | 1.44 | 1.51 | 0.46 |
| PE(36:5) | 0.10 | 0.05 | 0.52 | 0.11 | 0.05 | 0.01 | 0.27 | 0.03 |
| PI(36:5) | 0.03 | 0.01 | 0.12 | 0.03 | 0.03 | 0.01 | 0.06 | 0.03 |
| PG(36:5) | 0.00 | 0.00 | 0.02 | 0.02 | 0.00 | 0.00 | 0.04 | 0.02 |
| LPE(18:3) | 0.00 | 0.00 | 0.01 | 0.02 | 0.00 | 0.00 | 0.01 | 0.01 |
| MGDG(36:6) | 65.51 | 4.48 | 11.76 | 1.31 | 41.20 | 7.72 | 0.47 | 0.31 |
| DGDG(36:6) | 15.93 | 3.32 | 3.74 | 0.54 | 13.14 | 2.03 | 0.26 | 0.17 |
| PC(36:6) | 0.09 | 0.06 | 0.15 | 0.29 | 0.00 | 0.00 | 0.31 | 0.43 |
| PE(36:6) | 0.02 | 0.02 | 0.01 | 0.01 | 0.01 | 0.01 | 0.01 | 0.02 |
| PG(36:6) | 0.00 | 0.01 | 0.00 | 0.01 | 0.00 | 0.00 | 0.00 | 0.00 |
| PI(36:6) | 0.14 | 0.07 | 0.00 | 0.01 | 0.12 | 0.04 | 0.00 | 0.00 |
| PS(36:6) | 0.00 | 0.00 | 0.00 | 0.00 | 0.00 | 0.00 | 0.00 | 0.00 |
| PA(36:6) | 0.00 | 0.00 | 0.00 | 0.00 | 0.00 | 0.00 | 0.00 | 0.00 |
| PC(34:2) | 0.60 | 0.17 | 11.23 | 1.82 | 0.44 | 0.27 | 10.02 | 2.01 |
| PE(34:2) | 0.29 | 0.06 | 4.43 | 0.65 | 0.23 | 0.04 | 4.04 | 0.65 |
| PI(34:2) | 0.37 | 0.07 | 4.28 | 0.95 | 0.39 | 0.06 | 4.26 | 0.42 |
|  |  |  |  |  |  |  |  |  |
| 18:3/18:2 Ratio | 13.43 |  | 0.62 |  | 2.48 |  | 0.08 |  |

The youngest matured leaf blade and panicles ~3 cm long were sampled and analyzed for lipid content. 36:6 represents lipids with two linolenic acid moieties; 36:4, lipids with two linoleic acid moieties; and 36:5, lipids with one linolenic acid and one linoleic acid.

**Table 4.** Grain weight (mg/grain) before and after JA treatment.

| **Treatment** | **Grain weight** |  |
| --- | --- | --- |
| BTx623 | 3.0 |  |
| *Msd3-3* | 2.3 ** |  |
| BTx623-JA | 3.1 |  |
| *msd3-3*-JA | 3.1 |  |

Grain weight was determined from plants treated with 0.05% tween-20 or 0.05% Tween-20 containing 1 mM MeJA. Three samples of 100 grains were weighed from each treatment. Only the untreated *msd3-3* grain weight was significantly different from the wild type BTx623 and the JA-treated *msd3-3* mutants (p-value = 5.9 × 10^−7^).
